# A Coronal Landmark for Tibial Component Positioning With Anatomical Alignment in Total Knee Arthroplasty: A Radiological and Clinical Study

**DOI:** 10.3389/fsurg.2022.847987

**Published:** 2022-03-29

**Authors:** Tianlun Gong, Ruoyu Wang, Song Gong, Lizhi Han, Yihu Yi, Yuxiang Wang, Weihua Xu

**Affiliations:** Department of Orthopaedics, Union Hospital, Tongji Medical College, Huazhong University of Science and Technology, Wuhan, China

**Keywords:** mechanical alignment, lateral point of articular surface of distal tibia, total knee arthroplasty, tibial resection, anatomical alignment

## Abstract

**Objective:**

The purpose of this study was to investigate the value of the lateral point of articular surface of distal tibia (LADT) for anatomical alignment in total knee arthroplasty.

**Methods:**

We reconstructed 148 three-dimensional pre-arthritic tibias and measured the tibial component inclination angle corresponding to the distal landmark of LADT. A retrospective study included 81 TKA recipients divided into the AA group and MA group. Clinical assessments including ROM, HSS, WOMAC, satisfaction for surgery, and radiological assessment were evaluated at one-year follow-up.

**Results:**

The tibial component varus angle corresponding to the distal landmark of LADT in the male and female groups were 3.4 ± 0.3° (2.6~4.2°) and 3.2 ± 0.3° (2.3~4.0°), respectively (*P* <0.05). Using LADT as the distal landmark for extramedullary tibial cutting guidance, the medial proximal tibia angle (MPTA) of the AA group was 87.0±1.2° (85.0~90.0°), and the AA and MA technique showed no difference in improvement in postoperative knee functional recovery at final follow-up.

**Conclusions:**

This study preliminarily indicated that LADT can be a reliable and economical landmark for coronal plane alignment of the tibial component.

## Introduction

Total knee arthroplasty (TKA) is a surgery to replace eroded cartilage and bone with artificial implants. Correct alignment and proper balance are closely related to long-term implant survivorship and postoperative functional recovery. According to the position of the femoral and tibial components on the coronal plane, multiple philosophies for lower limb alignment have been developed, including the classic mechanical alignment (MA) (1) and anatomical alignment (AA) (2). MA was initially proposed by Insall et al. in the 1970s and is still the mainstream option for TKA (3). MA is performed by osteotomy perpendicular to the mechanical axis of the femur and tibia, respectively, to restore a neutral lower limb alignment. It is a kind of “systematic alignment” that has proven to be a reliable technique to reproduce a stable knee and a neutrally aligned lower limb. However, residual symptoms following MA-TKAs remain troublesome. Up to 20% of MA-TKA recipients are dissatisfied (4) and over half have residual symptoms, the main reasons include residual postoperative pain, joint stiffness, grinding or other noise, swelling or tightness, and expectations not met and less functional improvement (5).

AA was introduced by Hungerford and Krackow in 1980s (2) to improve postoperative functional recovery by preserving the original knee anatomy. Although AA still aims for a neutral HKA angle as MA, the bone cuts on the coronal plane are 3° oblique to their mechanical axis, respectively, to reflect the population's mean native joint line orientation (3° femoral valgus and 3° tibial varus) (6). Compared with MA, AA provides for a joint line parallel to the ground during normal gait (2). Bellemans et al. analyzed the lower limb force lines of 250 asymptomatic adult volunteers and found that for people with neutral lower limb alignment (hip-knee-ankle angle, HKA ± 3°), tibial plateau inclination was about 3° varus (7). For these patients, preservation of “normal” varus angle of the tibia may help to achieve optimal knee balance and improve postoperative functional recovery.

Tibial resection and modification of medial proximal tibial angle (MPTA) always take precedence over femoral resection when adjustment of lower limb alignment is required. Therefore, accurate tibial resection is essential for AA. There is always a risk of excessive varus resection when performing a 3° varus osteotomy with conventional instruments. And some authors worried that varus cut greater than 3° in the proximal tibia may lead to early failure of TKA or affect the service life of the tibial component (8–10).

At present, the commonly used techniques to improve surgical accuracy include computer navigation (11, 12), patient-specific cutting guides (13, 14), and robotics (15, 16). However, previous studies on the cost-effectiveness of these techniques have shown that either technique will increase the cost of each case, which mainly comes from the purchase and maintenance of machinery and equipment, additional preoperative imaging examinations, and limited implant choice of some closed robotic platform. In addition, the life span of the equipment and the hospital volume also has an impact. For medical centers with lower volume of joint replacements, the cost allocated to each case will be more significant (17–19). Without these technologies, some surgeons may estimate the varus degree of tibial resection according to the distance of the outward deviation of the extramedullary alignment rod. Due to individual variability in tibia length, this subjective method relies on personal experience may cause excessive varus tibial resection. The lack of a simple and effective method to determine the varus degree of tibial resection may be partly responsible for limiting the application of the AA technique. The purpose of this study was to propose that the lateral point of articular surface of distal tibia (LADT) can be used as a reliable landmark for varus tibia resection and to preserve some varus stably on the tibial component in TKA.

## Methods

### Pre-arthritic Tibia Measurements

The first part aims to measure the tibial component inclination angle corresponding to the lateral point of articular surface of distal tibia (LADT). Computer tomography (CT) data of patients who underwent angiography for lower extremity vascular disease were retrieved from the imaging database of Wuhan Union Hospital, China. The inclusion criteria followed two principles: (1) age ranged from 20 to 50 years; (2) Kellgren-Lawrence (K-L) grade 0 and grade I. The participants with altered skeletal structure of lower limbs were excluded. Considering the differences in the physiologic dimensions of the tibia between different genders, we performed statistical analysis by gender. Of the 82 participants (43 males and 39 females) who met the inclusion criteria, 3 were excluded for skeletal structure changes (1 male and 2 females). Finally, 37 females were enrolled in the study, 37 males were matched in a 1:1 paring, a total of 74 participants (148 tibias) CT data saved in DICOM format were enrolled.

**Figure 1:**
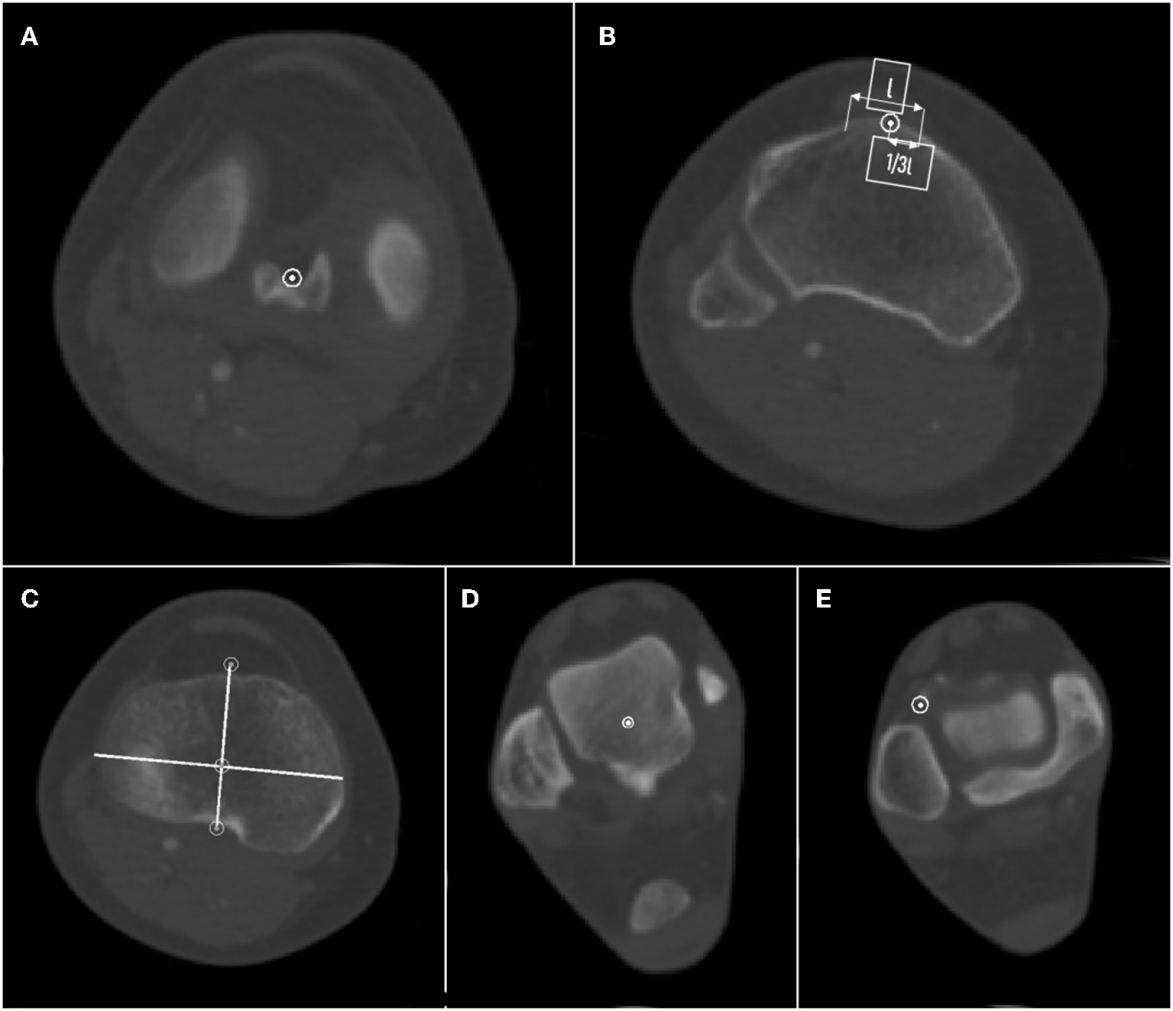
Schematic diagram of tibia measurement. **(A)** Tibial plateau center. **(B)** The medial third of the tibial tuberosity. **(C)** AP axis and transverse diameter of tibial plateau. **(D)** Ankle center. **(E)** Lateral point of articular surface of distal tibia (LADT). The arrow means tibial tuberosity width.

**Figure 2:**
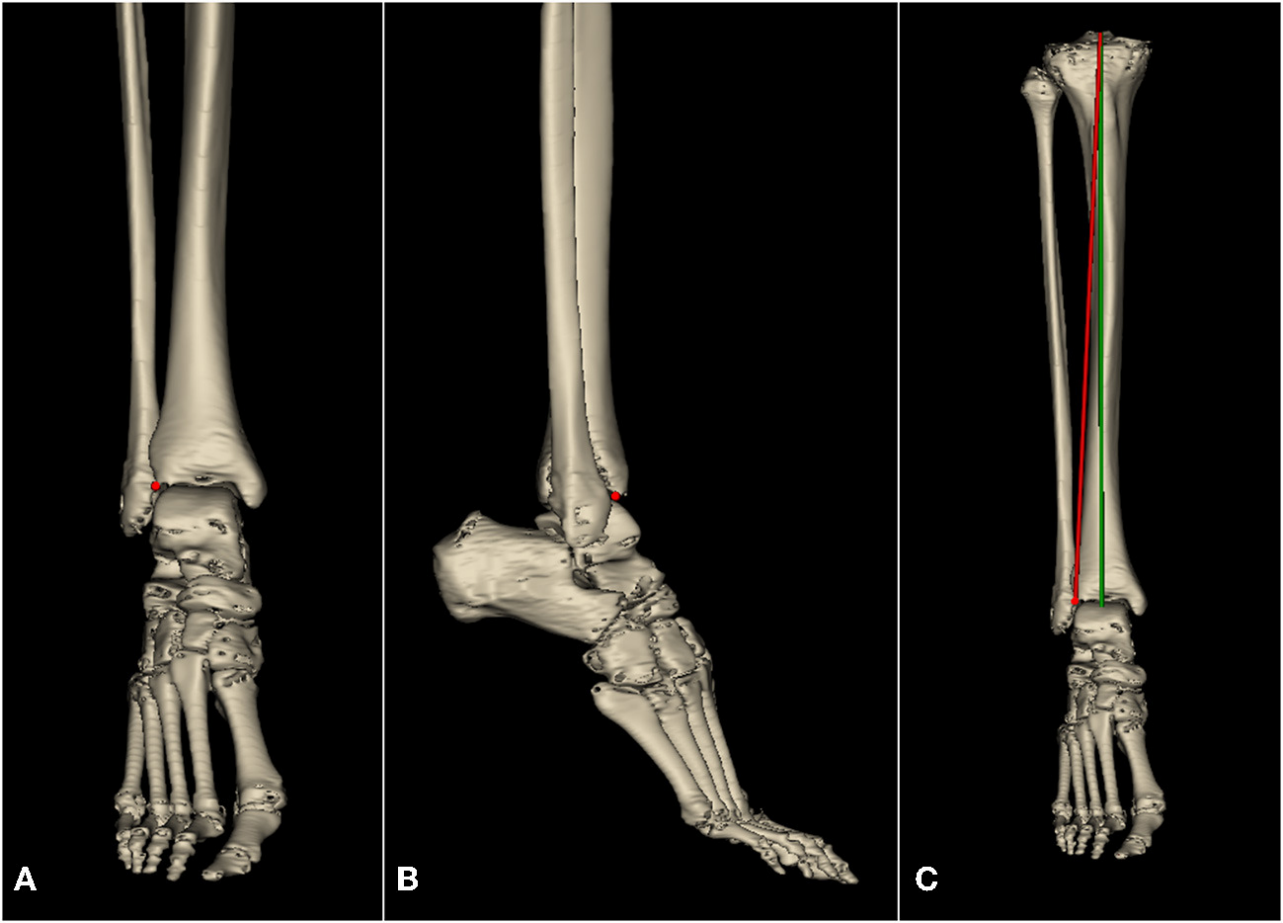
Lateral point of articular surface of distal tibia (LADT, red point) on the tibial reconstruction, and tibial component alignment of AA and MA. **(A)** Coronal plane. **(B)** Sagittal plane. **(C)** Tibial component alignment of AA (red line) and MA (green line).

CT data of 148 pre-arthritic tibias were loaded into Mimics Research 21.0 (Materialize Inc, Belgium) to regenerate lower extremity skeletal structures. The knee joint center was defined as the midpoint of the tibial intercondylar eminence, the ankle joint center was defined as the midpoint of the trochlea tali, and the tibial mechanical axis was a line connecting these two points. The femoral mechanical axis was a line connecting the center of the femoral head and the center of the knee joint. HKA was defined as the angle between the mechanical axis of the femur and the mechanical axis of the tibia, with both lines crossing at the center of the knee (20). The anteroposterior (AP) axis was the line drawn from the medial third of the tibial tuberosity to the mid-point of the posterior cruciate ligament. Pointed the LADT on the CT axial plane (Figure 1). The plane formed by the transverse axis of the tibial plateau and the midpoint of the tibiotalar joint was defined as the coronal plane, and the tibia, reference points, and reference lines were projected onto this plane for subsequent measurements. The line connecting the knee joint center and LADT and the tibial mechanical axis on the three-dimensional tibial model were shown in Figure 2. The angle between these two lines on the coronal plane was defined as the tibial anatomic mechanical angle (TAMA). When LADT was used as a distal landmark for coronal tibial component alignment, TAMA meant the expected varus inclination of the tibial component. The lateral distal femoral angle (LDFA) was defined as the angle formed by a line marking the most distal part of the femoral subarticular bone and the femoral mechanical axis. The MPTA was defined as the angle formed by a line marking the proximal extent of the tibial subarticular bone and the tibial mechanical axis (21). The transverse axis of the tibial plateau is a line through the knee center, parallel to the anterior border of the tibial plateau in the transverse plane, and the width of the tibia is the length of the transverse axis. The long axis of the tibia is the line running through the knee center and the center of the ankle, and the length of the tibia is the length of the long axis (22).

The statistical indicators of the first part of the study include age, gender, length and width of the tibia, the HKA, MPTA, and TAMA. All statistical analyses were performed using R 4.0.4. The Shapiro-Wilk normality test was used to measure the normal distribution of continuous variances. The continuous variables in this part included tibia length, tibia plate width, HKA, MPTA, TAMA. Independent *t*-test was used to compare means between groups by gender. A significant difference was defined as *P* < 0.05.

### Retrospective Clinical Study

The second part was a retrospective clinical study to examine the reference value of LADT as a distal landmark for proximal varus tibial resection in TKA. Electronic medical records were retrospectively reviewed. The patients undergoing TKA in Wuhan Union Hospital, China from 2019 to 2020 were divided into the AA group and MA group according to surgical records. The inclusion criteria were primary TKA due to osteoarthritis. The exclusion criteria were as follows: (1) musculoskeletal structural changes of lower limb due to any cause. (2) standing full-leg radiographs do not meet the standard (15° inward buckle of both feet and patella facing forward). Among the 107 participants who met the inclusion criteria, two had a history of lower limb fracture, 4 did not meet the standard of postoperative standing full-leg radiographs, and 20 were excluded for incomplete medical records or poor follow-up. Finally, 81 patients were enrolled in the study. All the TKA surgeries including AA and MA methods are performed by the same surgeon.

**Figure 3:**
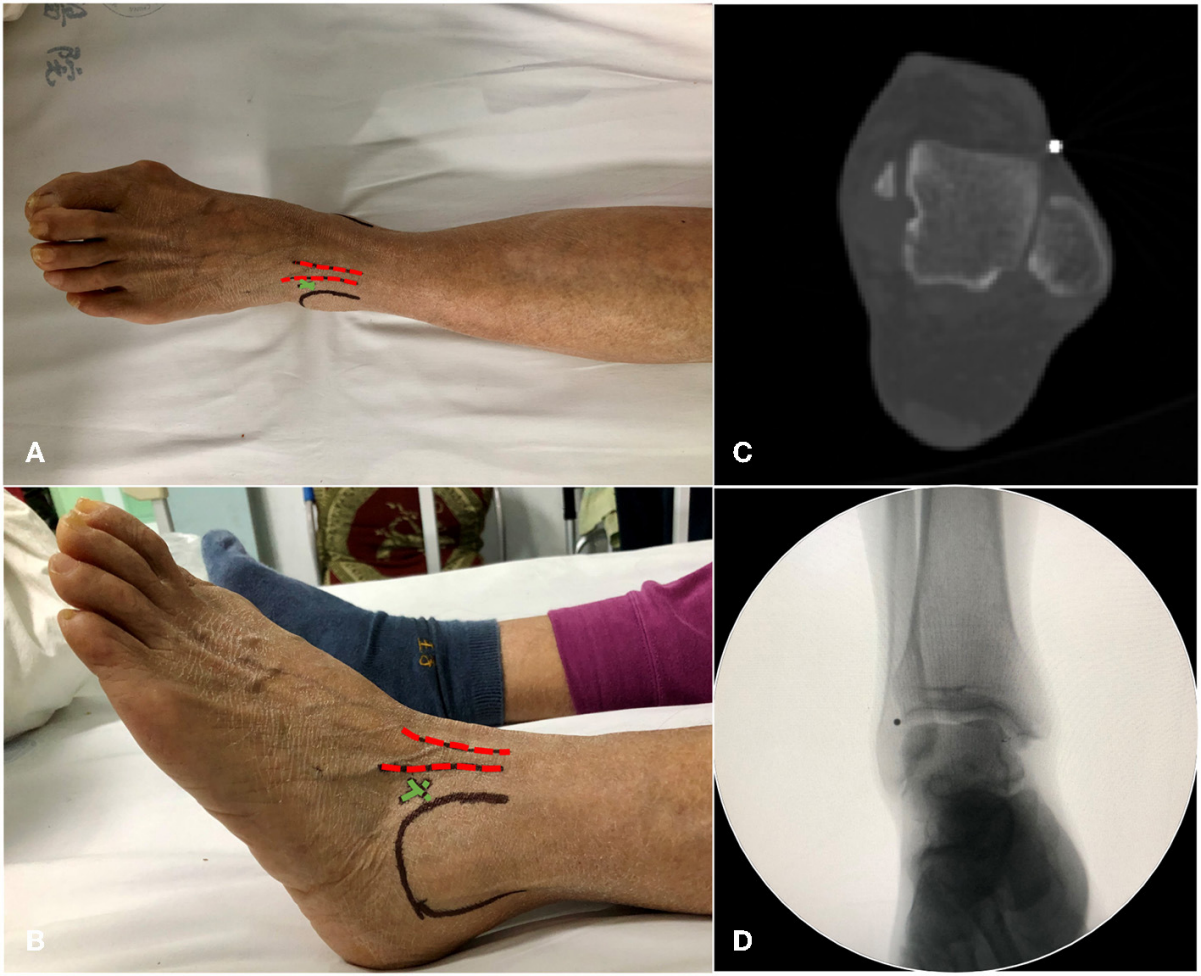
Schematic diagram of LDAT positioning. **(A,B)** Top and left views. Red dotted line, the extensor digitorum longus tendon; green cross, the LADT position; black circle, the malleolus lateralis. **(C,D)** Positioning LADT with a steel ball on the body surface, CT axial plane image and X-ray fluoroscopy image.

The retrospective study included 81 knee arthroplasty recipients. A standard incision with medial parapatellar arthrotomy was performed, tibial extramedullary guidance was used in all cases. In the second part of this study, the measurement methods for HKA, MPTA, LDFA, TAMA were the same as in the first part of the study. For the AA group, the surgeon estimated the “normal” varus angle of the operated tibia with reference to the unaffected knee preoperatively and measured TAMA on standing full-leg radiographs. During the surgery, the varus degree of the tibial component was determined by aligning the end of extramedullary rod with the LADT. The joint line orientation was restored to approximately 3° varus, the lower extremity alignment was rectified to within a neutral ±3° range. The LADT was positioned lateral to the ankle joint space, and the lateral depression of the common tendon of the extensor digitorum longus. A steel ball was placed at the LADT to verify the accuracy of body surface location by CT and fluoroscopy (Figure 3). For the MA group, TKA was performed according to the standard surgical procedures, the tibial resection was performed perpendicular to the mechanical axis.

Patient demographics of the second part of this study and body mass index (BMI) were recorded preoperatively. All patients were evaluated preoperatively and at follow-up visits using the range of motion (ROM) assessment, Hospital for Special Surgery (HSS), Western Ontario and McMaster Universities (WOMAC) scoring systems, and Likert score. The Likert score ranged from 2 to 5 and was used to evaluate patient satisfaction for surgery, respectively indicating dissatisfied, neutral, satisfied, and very satisfied. Radiographic evaluations were performed perioperatively, preoperative measures included TAMA, HKA, MPTA, LDFA, and postoperative measures included HKA, MPTA, LDFA, and tibial component varus angle.

All statistical analyses were performed using R 4.0.4. The Shapiro-Wilk normality test was used to measure the normal distribution of continuous variances. Two-way repeated measures ANOVA was used to analyze radiographic parameters of different surgical methods (AA or MA) and different surgical statuses (pre or postoperative) of each group. The continuous variables in this part included HKA, MPTA, LDFA, TAMA, tibial component varus angle, and ROM. Independent *t*-test was used in the comparison of continuous variables and preoperative minus postoperative values between groups by surgical method. The paired sample *t*-test was used to compare preoperatively measured TAMA and the postoperative tibial component varus angle in the second part of the study. The chi-square test was used for the comparison of gender distribution. Wilcoxon signed-rank test was used to analyze the non-continuous variables including the HSS score, WOMAC score, and Likert score. A significant difference was defined as *P* < 0.05.

The Ethics Committee of Tongji Medical College, Huazhong University of Science and Technology, approved this retrospective study. No patients received additional X-ray examinations or incurred additional medical expenses for this research.

## Results

### Demographics of Participants

Part I of the study included 74 participants, with an equal number of males and females. No statistical difference in age was observed between groups (Table 1). Part II of the study included 81 TKA recipients, 41 in the AA group and 40 in the MA group. No significant differences in preoperative patient demographics (including age, BMI, and sex ratio) between the AA and MA groups (Table 2).

**Table 1:** Demographic data and measurement of non-arthritic Tibia.

	**Male**	**Female**	***P*** **-value**
Cases (Tibias)	37 (74)	37 (74)	NA
Age (year)	39.8 ± 8.8 (23~50)	39.4 ± 7.4 (22~50)	0.762
Tibial length (mm)	353.0 ± 19.9 (305.8~384.9)	331.1 ± 15.6 (297.0~367.2)	0.000*
Tibial plate width (mm)	77.5 ± 3.6 (67.25~84.72)	69.3 ± 3.0 (60.2~76.4)	0.000*
HKA (°)	178.7 ± 1.9 (173.4~182.1)	179.3 ± 2.5 (173.2~186.0)	0.093
MPTA (°)	86.2 ± 2.0 (81.9~91.4)	87.6 ± 2.0 (83.3~94.6)	0.000*
TAMA (°)	3.4 ± 0.3 (2.6~4.2)	3.2 ± 0.3 (2.3~4.0)	0.000*

*Data presented as mean ± standard deviation (min~max)*.

*DHKA, hip-knee-ankle angle; MPTA, medial proximal tibia angle; TAMA, tibial anatomic mechanical angle*.

* *P < 0.05. NA, not applicable*.

**Table 2:** Preoperative demographic data.

	**AA**	**MA**	***P*** **-value**
Cases	41	40	NA
Age (years)	64.8 ± 8.0 (28~83)	64.7 ± 6.6 (52~79)	0.925
Gender (male/female)	9/41	9/40	0.953
BMI (kg/m^2^)	25.6 ± 3.5 (19.2~33.2)	25.7 ± 3.0 (17.7~31.2)	0.891

*Data presented as mean ± standard deviation (min~max); The chi-square test was used for the comparison of gender distribution and the independent sample t test was used for the remaining variables*.

*NA, not applicable*.

### Pre-arthritic Tibia Measurement

A total of 148 pre-arthritic tibias were enrolled. The measured tibia parameters were shown in Table 1. The differences in tibia length and tibial plateau width were compatible with the physiological conditions between genders. Both males and females have mild knee varus, with no statistical difference. The TAMA of the total population was 3.3 ± 0.3°, the maximum values in the male and female groups were 4.2° and 4.0°, and the mean values were 3.4 ± 0.3° and 3.2 ± 0.3°, respectively, *P* < 0.05 (Table 1).

### Radiological and Clinical Assessment

**Figure 4:**
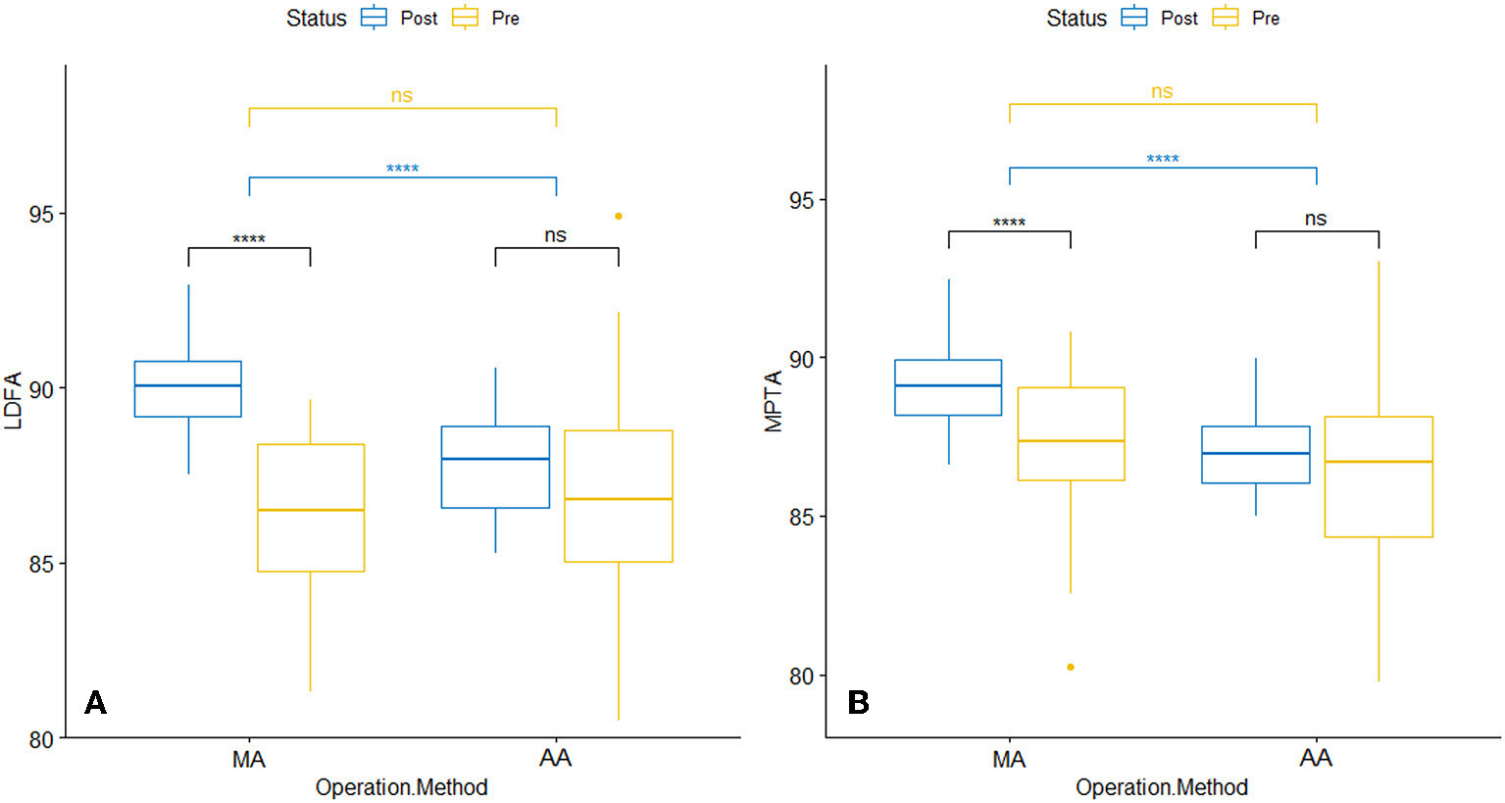
Perioperative radiographic parameters of different surgical groups. **(A)** LDFA. **(B)** MPTA. LDFA, lateral distal femoral angle; MPTA, medial proximal tibia angle; ****p < 0.0001, ns, not significant.

**Figure 5:**
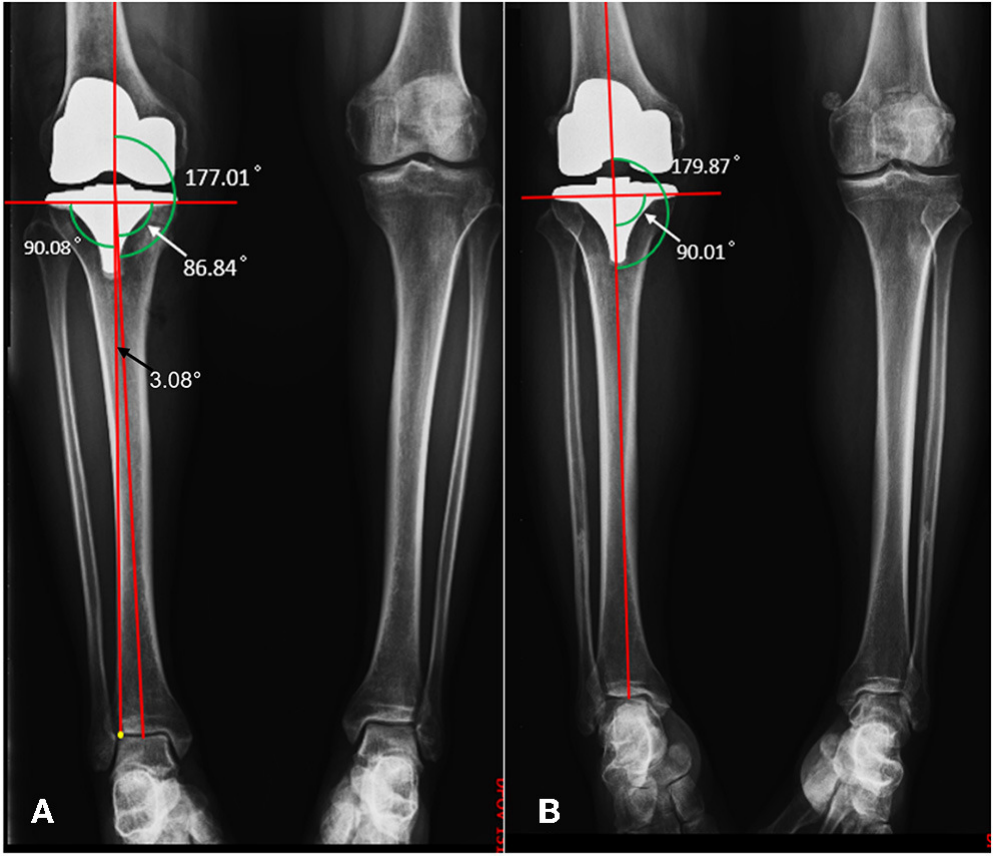
Postoperative radiological images of AA **(A)** and MA **(B)**. MPTA, white arrow; TAMA, black arrow; LADT, yellow point.

In terms of preoperative radiological and clinical assessment, including HKA, LDFA, MPTA, ROM, HSS, and WOMAC scores, no statistical difference was observed between the AA and the MA group (Table 3). The lower limb alignment of both groups was rectified through surgery, and there was no significant difference in postoperative HKA. The average postoperative MPTA of the AA and MA groups were 87.0 ± 1.2° and 89.1 ± 1.4° respectively, *P* < 0.05. Compared with the MA group, there was no statistical difference in LDFA and MPTA before and after surgery in the AA group, indicating that the AA technique had less alteration in the native knee anatomy and kinematics (Figure 4). The preoperative TAMA measured in the AA group was 2.9 ± 0.3° (2.3°~3.8°), and the postoperative tibial component varus angle was 3.0 ± 1.2° (0.02°~5.0°), *P* = 0.51, with no significance, indicating that the method of LADT achieves the expected surgical goals. The absolute values of perioperative HKA changes in the AA and the MA groups were 6.4 ± 3.7° and 4.8 ± 3.0° respectively, *P* < 0.05, indicating more correction of lower limb alignment in the AA group. And the changes in LDFA and MPTA in the AA group were also smaller than those in the MA group, indicating that AA had fewer changes in the inherent anatomy of the knee joint (Table 4). Typical case examples of both alignment techniques were shown in Figure 5. For the patient who received AA-TKA (5a), the tibial component had 3.2° varus. The line connecting the knee joint center and the LADT (yellow point) was almost perpendicular to the tibial joint line orientation (90.1°), and the HKA angle was 177.0°.

**Table 3:** Perioperative radiological and clinical assessment.

		**AA**	**MA**	***P*** **-value**
HKA (°)	Pre	176.1 ± 7.5 (163.7~191.5)	177.1 ± 5.5 (169.0~192.5)	0.517
	Post	179.1 ± 1.5 (177.0~182.2)	179.7 ± 1.4 (177.1~182.8)	0.093
LDFA (°)	Pre	86.9 ± 3.2 (80.5~94.9)	86.5 ± 2.1 (81.3~89.6)	0.458
	Post	87.9 ± 1.5 (85.3~90.6)	89.9 ± 1.3 (87.5~92.9)	0.000*
MPTA (°)	Pre	86.3 ± 2.9 (79.8~93.0)	87.2 ± 2.3 (80.3~90.8)	0.134
	Post	87.0 ± 1.2 (85.0~90.0)	89.1 ± 1.4 (86.6~92.5)	0.000*
ROM (°)	Pre	105.8 ± 5.2 (96~118)	104.5 ± 5.4 (95~115)	0.287
	Post	118.6 ± 3.2 (111~126)	117.9 ± 3.0 (113~124)	0.289
HSS	Pre	56.3 ± 2.8 (51~62)	56.3 ± 2.8 (52~62)	0.928
	Post	90.1 ± 2.2 (84~94)	90.0 ± 2.4 (85~94)	0.030
WOMAC	Pre	59.7 ± 3.7 (54~69)	59.3 ± 3.0 (55~67)	0.588
	Post	20.1 ± 2.4 (16~26)	21.2 ± 2.2 (16~24)	0.024
Likert score	Post	4.3 ± 0.7 (3~5)	3.8 ± 0.7 (2~5)	0.003*

*Data presented as mean ± standard deviation (min~max); ROM, range of motion; HSS, hospital for special surgery score; Womac Western Ontario and McMaster Universities Osteoarthritis Index*.

* *P < 0.05*.

**Table 4:** Comparison of preoperative minus postoperative values.

		**AA**	**MA**	***P*–value**
HKA (°)	RD	−3.0 ± 6.8 (−13.8~12.5)	−2.6 ± 5.1 (−10.1~10.6)	0.767
	AD	6.4 ± 3.7 (0.1~13.8)	4.8 ± 3.0 (0~10.6)	0.034*
LDFA (°)	RD	−1.0 ± 2.8 (−8.4~7.0)	−3.4 ± 2.0 (−9.3~0.2)	0.000*
	AD	2.2 ± 1.9 (0.1~8.4)	3.5 ± 2.0 (0.2~9.3)	0.006*
MPTA (°)	RD	−0.7 ± 2.7 (−5.8~6.2)	−1.9 ± 2.6 (−7.1~3.2)	0.045*
	AD	2.2 ± 1.7 (0.2~6.2)	2.6 ± 1.9 (0.02~7.1)	0.370
ROM (°)	RD	−12.8 ± 4.0 (−24~-5)	−13.4 ± 4.0 (−24~-7)	0.507
	AD	12.8 ± 4.0 (5~24)	13.4 ± 4.0 (7~24)	0.507
HSS	RD	−33.8 ± 3.2 (−40~-28)	−32.6 ± 3.7 (−40~-24)	0.170
	AD	33.8 ± 3.2 (28~40)	32.6 ± 3.7 (24~40)	0.170
WOMAC	RD	39.6 ± 3.8 (33~49)	38.1 ± 2.9 (33~45)	0.067
	AD	39.6 ± 3.8 (33~49)	38.1 ± 2.9 (33~45)	0.067

*Data presented as mean ± standard deviation (min~max)*.

*RD, real data; AD, absolute data*.

* *P < 0.05*.

At the final follow-up, there was no significant difference in ROM but significant differences in HSS and WOMAC scores (Table 3). However, the comparison of preoperative minus postoperative values indicated that there was no statistical difference in the changes of HSS, WOMAC and ROM between the two groups (Table 4), which meant AA and MA showed no difference in postoperative recovery. And the AA group expressed a higher level of satisfaction with their surgeries (Table 3).

## Discussion

In this study, we found that over 54% of tibial joint lines had 3° or more varus inclination, and the MPTA was 86.2 ± 2.0° (male) and 87.6 ± 2.0° (female). The TAMA of the total population was 3.3 ± 0.3°, the maximum values in the male and female groups were 4.2° and 4.0° respectively, and the mean values were 3.4 ± 0.3° and 3.2 ± 0.3°. A retrospective study of 81 TKA recipients found that the postoperative MPTA was 87.0 ± 1.2° (85.0°~90.0°) in the AA group using LADT as a landmark for extramedullary tibial cutting guide, while the postoperative MPTA in the MA group was 89.1 ± 1.4° (86.6°~92.5°), *P* < 0.05, indicating that our technique facilitates accurate varus tibial resection. Postoperative HKA was restored to near neutral alignment in both groups, 179.1 ± 1.5° (177.0°~182.2°) in the AA group and 179.7 ± 1.4° (177.1°~182.8°) in the MA group, with no statistical difference. In the AA group, there was no significant difference in preoperative TAMA and postoperative tibial component varus angle. The 1-year follow-up results showed that there was no significant difference in the improvement of ROM, HSS score and WOMAC scores between AA and MA, while the patient satisfaction in the AA group was better.

When TKA was initially introduced in the 1970s, many authors believed that the restoration of the neutral axis is important for successful clinical outcomes and implant survivorship (23, 24). Thus, the MA technique was introduced to standardize the procedure and ensure implant survivorship, rather than reproducing normal knee anatomy and function. However, when performing MA-TKAs, non-anatomical bone cuts may cause mediolateral and flexion-extension joint gap imbalances and thus alter the patella tracking, quadriceps function, and ultimately the knee kinematics (25–27). These issues have not been solved by advanced technologies, thus indicating intrinsic limitations of the MA technique (28, 29). It may be unreasonable to reproduce neutral mechanical alignment in patients with “normal” tibial varus angle.

Unlike the “one size fits all” approach of MA, in TKA using the AA technique, the natural joint line orientation (average 3° varus) is preserved to reduce alterations to knee kinematics, thereby improving postoperative functional recovery and patient satisfaction. The rational support of AA is that it better simulates the physiology of load distribution on the tibial component (30) and reduces the risk of lateral retinacular ligament stretching when the knee flexes, so as to achieve better patellar biomechanics (31). Our study found that although the postoperative MPTA of the AA group was smaller than that of the MA group, and the real MPTA change in the AA group was also smaller, there was no significant difference in the absolute MPTA, which meant that the AA technique changed the patients' native anatomy to the same extent as the MA technique. This may explain why there was no significant difference in postoperative functional improvement between the two groups. To minimize anatomical changes caused by TKA, kinematic alignment (KA) (32) or restricted kinematic alignment (rKA) (33) combined with precise tools may be an alternative.

At present, there are not many comparative clinical studies on the mechanical and anatomical alignment methods. This may be due to the inability of conventional instrumentations to address the risk of excessive varus of the tibial component positioning. Ji-Hyun Yim et al. addressed the issue of bone cut accuracy with a TKA robot and compared AA and MA, and the results suggested that there was no statistical difference in clinical and radiological outcomes (34). Our results also confirmed this. In addition to robots, common assistive technologies include computer navigation and patient-specific cutting guides. There has been abundant evidence that computer navigation (35, 36) and robots (37, 38) can produce better precision and prosthesis alignment than conventional instrumentation but limited evidence that this translates into better long-term clinical outcomes. Patient-specific cutting guides also require customization. Therefore, either computer navigation, Patient-specific cutting guides, or robots will increase the pre-operative preparations and financial burden of patients.

To find a reliable reference landmark for AA, we hypothesized that the LADT can be used for the coronal alignment of the tibial component. The results of tibial measurements and retrospective clinical study confirmed our speculation. As for the accuracy of this method, compared with the TAMA angle obtained from tibial measurement (standard deviation 0.3°) in the first part of this study, the postoperative tibial component varus angle in the AA group fluctuated more (standard deviation 1.2°). This may be caused by the obstruction of skin and soft tissue, resulting in some deviation in the location of the LADT, and this impact may be more pronounced in obese people. After confirmation of the accuracy of LADT in the first part, in the second part of this study, the preoperative TAMA and the postoperative tibial component varus angle in the AA group did not show a significant difference, the minimum value of postoperative MPTA was 85.0°, confirming that the method of LADT can achieve the desired surgical goals. Compared with computer navigation, patient-specific cutting guides, and robots, the LADT can be a complementary method with acceptable accuracy.

Certain limitations of the present study should be mentioned. The accuracy of the LADT is high from CT measurements, but its performance may be compromised in practical applications. How to locate this point more accurately in surgery requires further investigation. Secondly, for the clinical assessment, the sample size and the follow-up time were inadequate. The one-year follow-up is not enough to observe the difference in implant survivorship. More samples and extended follow-up can further enhance the persuasiveness of the results. Third, the clinical study is partially abbreviated and lacks gait analysis and knee biomechanics analysis, which needs further research in the future. Finally, this study was limited to the coronal plane, while the component is positioned in a three-dimensional space, further research is needed on how to combine the alignment of the other two dimensions with the varus alignment on the coronal plane.

## Conclusion

In conclusion, to solve the problem of replicating accurate varus tibial resection with conventional instrumentation, we proposed a reliable landmark, the LADT, for varus tibial resection on the coronal plane, and confirmed its reliability in surgery. The clinical follow-up suggested that AA technique improves patient satisfaction for surgery, but it has not yet shown advantages in improving functional recovery, and its impact on implant survivorship requires further follow-up.

## Data Availability Statement

The raw data supporting the conclusions of this article will be made available by the authors, without undue reservation.

## Ethics Statement

The studies involving human participants were reviewed and approved by Ethics Committee of Tongji Medical College, Huazhong University of Science and Technology. The patients/participants provided their written informed consent to participate in this study.

## Author Contributions

TG, RW, and WX are responsible for the integrity and authenticity of this work, conception and design, and writing and critical revision of the article. YY and YW: literature research. SG and LH: data extraction. TG and RW: data analysis. All authors have read and approved the final version of this manuscript submitted for publication.

## Conflict of Interest

The authors declare that the research was conducted in the absence of any commercial or financial relationships that could be construed as a potential conflict of interest.

## Publisher's Note

All claims expressed in this article are solely those of the authors and do not necessarily represent those of their affiliated organizations, or those of the publisher, the editors and the reviewers. Any product that may be evaluated in this article, or claim that may be made by its manufacturer, is not guaranteed or endorsed by the publisher.

## Abbreviations

MA: mechanical alignment
AA: anatomical alignment
TKA: total knee arthroplasty
LADT: lateral point of articular surface of distal tibia
HKA: hip-knee-ankle angle
MPTA: medial proximal tibial angle
LDFA: lateral distal femoral angle
TAMA: tibial anatomic mechanical angle.
