# Corrigendum: A Coronal Landmark for Tibial Component Positioning With Anatomical Alignment in Total Knee Arthroplasty: A Radiological and Clinical Study

**DOI:** 10.3389/fsurg.2022.938517

**Published:** 2022-06-17

**Authors:** Tianlun Gong, Ruoyu Wang, Song Gong, Lizhi Han, Yihu Yi, Yuxiang Wang, Weihua Xu

**Affiliations:** Department of Orthopaedics, Union Hospital, Tongji Medical College, Huazhong University of Science and Technology, Wuhan, China

**Keywords:** mechanical alignment, lateral point of articular surface of distal tibia, total knee arthroplasty, tibial resection, anatomical alignment

**A Corrigendum on**

**A Coronal Landmark for Tibial Component Positioning With Anatomical Alignment in Total Knee Arthroplasty: A Radiological and Clinical Study**

*by Gong, T., Wang, R., Gong, S., Han, L., Yi, Y., Wang, Y., et al. (2022). Front. Surg. 9:847987. doi: 10.3389/fsurg.2022.847987*

**Incorrect Affiliation**

In the published article, there was an error in affiliation **1**. Instead of “** Department of Orthopaedics, Tongji Medical College, Union Hospital, Huazhong University of Science and Technology, Wuhan, China**”, it should be “** Department of Orthopaedics, Union Hospital, Tongji Medical College, Huazhong University of Science and Technology, Wuhan, China **”. The authors apologize for this error and state that this does not change the scientific conclusions of the article in any way. The original article has been updated.

## Conflict of Interest

The authors declare that the research was conducted in the absence of any commercial or financial relationships that could be construed as a potential conflict of interest.

## Publisher's Note

All claims expressed in this article are solely those of the authors and do not necessarily represent those of their affiliated organizations, or those of the publisher, the editors and the reviewers. Any product that may be evaluated in this article, or claim that may be made by its manufacturer, is not guaranteed or endorsed by the publisher.

